# Impact of second line limiting amino acids’ deficiency in broilers fed low protein diets with rapeseed meal and de-oiled rice bran

**DOI:** 10.14202/vetworld.2015.350-357

**Published:** 2015-03-18

**Authors:** C. Basavanta Kumar, R. G. Gloridoss, K. Chandrapal Singh, T. M. Prabhu, Siddaramanna, B. N. Suresh, G. A. Manegar

**Affiliations:** 1 Department of Animal Nutrition, Veterinary College, Hebbal, Bangalore, India; 2 Division of Animal Sciences, Karnataka Veterinary, Animal and Fisheries Sciences University, Hebbal, Bangalore, India; 3 Senior Technical Officer, Southern Research Station, National Dairy Research Institute, Bangalore, India; 4 Department of ILFC, Veterinary College, Hassan, India; 5 Department of Animal Science, Krishi Vignyana Kendra, Bangalore Rural Dist, India

**Keywords:** isoleucine, tryptophan and broilers, valine

## Abstract

**Aim:**

To study the impact of deficiency of second line limiting amino acids (SLAA; valine, isoleucine and tryptophan) on the production performance and carcass characteristics of commercial broilers.

**Materials and Methods:**

A control (T_1_) corn-soy diet was formulated to contain all essential AA on standardized ileal digestible basis; While in T_2_-a ‘moderate SLAA deficit’ diet was formulated by replacement of soybean meal with 6% rapeseed meal and T_3_-a ‘high SLAA deficit’ diet was formulated by replacement of soybean meal with 6% de-oiled rice bran. Each of these treatments was allotted to six replicates of ten chicks each. During the 42 days experimental period, growth performance, carcass parameters and intake of metabolizable energy (ME), crude protein (CP) and AA were studied.

**Results:**

The cumulative body weight gain, feed conversion ratio, carcass cut weights and yields of carcass, breast and thighs were decreased (p<0.05) in T_3_ compared to T_1_. The absolute intake of ME, lysine, methionine + cysteine and threonine were not affected while intake of CP and all SLAA were reduced in SLAA deficit diets. The relative intake of ME, lysine, methionine + cysteine, threonine and SLAA reduced in T_3_ in comparison to T_1_. The relative weights of internal organs were not affected by treatments while the abdominal fat percentage was increased linearly to the magnitude of SLAA deficiency.

**Conclusion:**

The deficiency of SLAA decreased performance, carcass yields and impaired utilization of ME, CP and AA linearly to the magnitude of the deficiency.

## Introduction

Protein is un-debatably an important nutrient for poultry and should be an integral part of the diet. Protein rich ingredients accounts for 30-40% of the ration yet play an imperative role in determining the feed cost. It is universally accepted that, birds as such do not have a need for crude protein (CP) rather there is a need for amino acids (AA). In commercial poultry feed formulations, the first line of limiting AA are supplemented extensively as DL-methionine, L-lysine and L-threonine to rationalize the AA levels and to economize feed cost on corn soy diets. Lowering the dietary CP levels and use of synthetic AA perhaps reduce the cost of diet and the environmental pollution of nitrogen [1]. Hence, reduction of protein through inclusion of locally available feed ingredients such as rape seed meal (RSM) and de oiled rice bran (DORB) can be thought of as an alternative solution.

In previous studies, the RSM was used up to a level of 6-10% of the ration [2-4]. Very similarly, although the DORB was included up to 20% in broiler rations [5,6], yet other reports [7,8] indicate deleterious effects of DORB on broiler performance. However, it is noteworthy to state that, none of the studies conducted with inclusion of either RSM or DORB addressed the issue of protein reduction, and moreover, the soybean meal content was adjusted to make the diets iso-nitrogenous. Furthermore, the level of L-lysine maintained in such studies was far below the level recommended for present day commercial broilers, which allowed safe use of these resources, without causing deficiency of third and subsequent limiting AA. Formulation of diets based on standardized ileal digestible AA was suggested as a means for better utilization of alternative feed ingredients [9]. However, in RSM and DORB based low CP diets formulated to meet the recommended ideal standardized ileal digestible (SID) AA ratio profile [10], valine, isoleucine and occasionally tryptophan were found to be second line limiting AA notwithstanding the supplementation of L-lysine, DL-methionine and L-threonine.

Since, CP reduction was a theme of this study, RSM and DORB were incorporated to reduce CP by replacing soybean meal on W/W basis (6%), and the resultant impact and gravity of second line limiting AA (SLAA) (valine, isoleucine and tryptophan) deficiency on the broiler performance, carcass characteristics and intake of nutrients were assessed in this study.

## Materials and Methods

### Ethical approval

Prior approval of Institutional Animal Ethics Committee was obtained for use of chicks and experimental procedures adopted in this study.

### Ingredients and AA analysis

Sufficient quantities of required feed ingredients were procured and analyzed for proximate principles [11]. Each ingredient was analyzed for AA composition at Evonik Laboratory. The feed formulation was done using the laboratory estimated CP and prorata AA composition of ingredients [12]. The AA profile of the experimental diets was arrived at based on the analyzed AA profile of each raw material.

### Experimental design and diets

A control (T_1_) broiler diet formulated to meet recommended ideal SID AA ratio [10] in such a way to meet the requirement of fourth limiting AA (valine/isoleucine depending on the feeding phase) under pre-starter (0-14 days), starter (15-28 days) and finisher (29-42 days) phases (Table-1). In the test diet T_2_ (moderate SLAA deficiency), soybean meal was replaced on W/W basis with 6% RSM and the levels of lysine, methionine + cysteine and threonine were maintained as in T_1_ with supplemental feed grade AA. Similarly, in T_3_ (high SLAA deficiency) soybean meal was replaced on W/W basis with 6% DORB and here also the levels of lysine, methionine + cysteine and threonine were maintained on par with control through supplementation. Diets were formulated to contain the same energy, calcium, and available phosphorus while the CP was allowed to dilute. The acid-base imbalance in low protein diets was corrected on par to control. The nutrient and AA composition of experimental diets is presented in Table-2. The magnitude of SLAA deficiency created with replacement of soybean meal by RSM and DORB in test diets is presented in Table-3.

**Table-1:** Ingredient composition of the experimental diets.

Ingredient (Kg/ton)	Pre-starter diet			Starter diet			Finisher diet		
		
	T_1_	T_2_	T_3_	T_1_	T_2_	T_3_	T_1_	T_2_	T_3_
Maize	484.5	475.5	473.0	537.0	527.5	524.5	597.0	587.5	585.0
Meat and bone meal	-	-	-	40.0	40.0	40.0	50.0	50.0	50.0
Rapeseed meal	-	60.0	-	-	60.0	-	-	60.0	-
DORB	-	-	60.0	-	-	60.0	-	-	60.0
Soybean meal	421	361	361	350.7	290.7	290.7	282.1	222.1	221.1
Dicalcium phosphate	20.0	20.0	20.0	7.5	7.5	7.5	4.5	4.5	4.5
Calcite powder	12.5	12.2	12.9	5.3	5.0	5.7	4.1	4.0	4.7
Salt	4.0	4.0	4.0	4.0	4.0	4.0	4.0	4.0	4.0
Soda bicarbonate	0.5	0.5	0.5	0.5	0.5	0.5	0.5	0.5	0.5
Trace mineral premix^1^	1.0	1.0	1.0	1.0	1.0	1.0	1.0	1.0	1.0
Vegetable oil	46.9	53.7	53.4	44.3	51.3	51.0	47.2	54.2	53.9
L-lysine monohydrochloride	0.78	1.66	2.79	0.98	1.86	2.98	1.02	1.90	3.03
DL-methionine	3.27	3.12	4.03	3.19	3.05	3.95	2.79	2.64	3.55
L-threonine	0.36	0.62	1.30	0.49	0.75	1.43	0.46	0.72	1.41
Potassium carbonate		1.05	0.73		1.08	0.76		1.07	0.74
Additives^2^	6.00	6.00	6.00	6.00	6.00	6.00	6.00	6.00	6.00
Total	1000.8	1000.4	1000.6	1000.7	1000.4	1000.3	1000.5	1000.6	1000.1

1 Contained Fe-90000 ppm, I–2000 ppm, Cu–15000 ppm, Mn–90000 ppm, Zn-80000 ppm, Se–300 ppm,

2 Contained vitamin A-10 mIU, D3-2.0 mIU, E-30.0 g, C-50 g, B_1_-2.0 g, B_2_-10.0 g, B_6_-3.0 g, B_12_-0.015, Niacin-30.0 g, calcium-d-pantothenate 15.0 g, biotin-0.10 g, folic acid- 2.0 g and vitamin-K-4.0 g; herbal liver stimulant-1700 g; semduramicin-30.0 g; tetracyclin-30.00 g; a commercial toxin binder-2000 g, DORB=De oiled rice bran

**Table-2:** Nutrient and amino acid composition of the experimental diets under different phases.

Ingredient (Kg/ton)	Pre-starter diet			Starter diet			Finisher diet		
		
	T_1_	T_2_	T_3_	T_1_	T_2_	T_3_	T_1_	T_2_	T_3_
CP (analyzed, %)	23.25	22.83	21.73	22.44	22.02	20.93	20.33	19.91	18.82
ME (Kcal/kg; calculated)	3000	3000	3000	3100	3100	3100	3200	3200	3200
Calcium %	1.01	1.01	1.01	0.91	0.91	0.91	0.89	0.90	0.89
P_av_%	0.47	0.47	0.48	0.45	0.45	0.46	0.44	0.45	0.45
Amino acid composition (as SID calculated from analyzed value*) (%)									
Lysine	1.204	1.205	1.205	1.120	1.120	1.120	0.983	0.983	0.983
Methionine+cystine	0.882	0.883	0.882	0.840	0.840	0.840	0.757	0.757	0.757
Threonine	0.772	0.773	0.773	0.728	0.728	0.728	0.649	0.649	0.649
Valine	0.953	0.924	0.838	0.896	0.851	0.779	0.803	0.763	0.688
Isoleucine	0.885	0.843	0.775	0.806	0.751	0.696	0.708	0.666	0.598
Tryptophan	0.240	0.233	0.209	0.211	0.205	0.180	0.180	0.173	0.149

* SID coefficients were taken from (3), SID=Standardized ileal digestible, CP=Crude protein, ME=Metabolizable energy

**Table-3:** The relative percentage of SID valine, isoleucine and tryptophan composition* as affected by inclusion of RSM and DORB.

Amino acid	Pre starter			Starter			Finisher		
		
	T_1_	T_2_	T_3_	T_1_	T_2_	T_3_	T_1_	T_2_	T_3_
Valine	100	97	88	100	95	87	101	98	87
Isoleucine	108	103	95	103	96	89	100	94	85
Tryptophan	125	121	108	118	114	100	108	104	89

T_1_: Control; T_2_: Moderate SLAA deficit diet; T_3_: High SLAA deficit diet,

* as compared to the ideal SID AA ratio recommended by (3), AA=Amino acid, SLAA=Second line limiting amino acids, SID=Standardized ileal digestible, DORB=De oiled rice bran, RSM=Rape seed meal

### Experimental birds

A total of 180 day-old straight run commercial broiler chicks were weighed, wing banded and divided into eighteen homogenous groups with ten chicks in each pen. The three experimental diets were randomly allocated to six pens each and each pen was considered as one experimental unit. All the chicks were reared in deep litter system in conventional open ventilated sheds with standard vaccination program and uniform managemental practices throughout the experiment.

### Parameters studied

Growth performance parameters: Feed intake and body weight of the individual birds in each replicate were recorded at weekly intervals. The mortality of the bird was recorded as and when occurred. The mortality corrected feed conversion ratio (FCR) was calculated as unit feed intake to the unit body weight gain (BWG).

Dressing percentage and organometry: Two birds from each replicate were randomly selected at the end of the trial (42^nd^ day), starved overnight with the provision for *ad lib* water and sacrificed by cervical dislocation. The dressing percentage was calculated as the percent of the carcass weight to the body weight after removing the feathers, neck, legs and internal viscera. Weight of different cuts *viz*., breast, thigh, drumstick and wing of the carcass was taken, and each part was expressed as a percent of pre-slaughter live weight (g/100 g). From each sacrificed bird, the weight of the giblet organs *viz*., heart (without pericardium), liver (without gall bladder) and gizzard (without inner layer) and additionally weight of abdominal fat was recorded and expressed as the per cent of pre-slaughter live weight (g/100 g).

Intake of ME, CP and AA: Based on the feed intake, intake of ME calculated based on the reported value while the CP and AA intakes were calculated based on the analyzed CP and AA composition.

### Statistical analysis

The experimental data were statistically analyzed by one-way ANOVA using means of six variants for each treatment. Tukey’s test with p<0.05 was used for mean separation wherever treatment effect was significant (p<0.05). Statistical analysis of data was performed using GraphPad Prism [13].

## Results

Inclusion of 6% RSM at pre-starter phase resulted in marginal deficiency (Table-3) of valine alone (3%) while at starter and finisher phase, both valine (5% and 2%) and isoleucine (4% and 6%) were found to be deficit. The DORB inclusion at pre-starter and starter phase resulted in both valine (12% and 13%) and isoleucine (5% and 11%) deficiency while at finisher phase, in addition to deficiency of valine (13%) and isoleucine (15%), deficiency of tryptophan (11%) was also evident. The results imply that deficiency of valine was much larger than that of isoleucine and tryptophan in that order during pre-starter and starter phase while it was almost similar during finisher phase.

The BWG (Table-4) of birds was found to be significantly affected by treatments at all stages of life. During pre-starter phase, BWG in T_3_ was significantly (p<0.05) reduced by 14.21% compared to that of T_1_, while T_2_ remained non-significant from both T_3_ and T_1_. However, the trend in the starter phase was quite different with significant (p<0.05) reduction of BWG in both T_2_ and T_3_ by 10.43 and 10.77% respectively from T_1_. However, in the finisher phase, only high SLAA deficiency resulted in significant (p<0.05) BWG reduction, but not the moderate deficiency group. On overall cumulative basis, the BWG in T_2_ (4.98%) and T_3_ (7.48%) were significantly (p<0.05) inferior to that of control and the response was found to be significantly (p<0.001) linear to the magnitude of SLAA deficiency. The feed intake (FI) in T_3_ during pre-starter phase was significantly (p<0.05) reduced (4.48%) in comparison to that of control and the FI in T_2_ was statistically similar (p≥0.05) to those of T_1_ and T_3_ groups whereas, in contrast, during starter phase, T_2_ was significantly (p<0.05) inferior to T_1_ and was non-significant with the T_3_ which stood in middle. However in contrary, during the finisher phase and on the cumulative basis there was a non-significant effect of treatments on the FI. The FCR (Table-4) was significantly (p<0.001) altered due to SLAA deficiency throughout the phases and cumulatively as well. FCR was significantly (p<0.05) different in each treatment during pre-starter and finisher phases. On the other hand, at starter phase, FCR in T_2_ and T_3_ was significantly (p<0.05) deprived than control despite of being non-significant from each other. On cumulative basis in T_2_ and T_3_, a significantly (p<0.05) poor FCR of magnitude 0.039 and 0.061 units was evident in contrast to control with a clear-cut linear effect of SLAA deficiency (p<0.001). The mortality rate was numerically high in T_3_ (5.00%) and T_2_ (3.33%) in comparison to T_1_ (2.00%) although it was statistically non-significant.

**Table-4:** Effect of second line limiting amino acid deficit diets on the BWG, feed intake and feed conversion ratio at different phases.

Treatment	Average BWG (g)				Average FI (g)				Average FCR (g per g gain)			
			
	Pre-starter	Starter	Finisher	Cumulative	Pre-starter	Starter	Finisher	Cumulative	Pre-starter	Starter	Finisher	Cumulative
T_1_	292.8^a^	763.5^a^	1071^a^	2127^a^	345.8^a^	1067^a^	2062	3475	1.181^a^	1.434^a^	1.967^a^	1.663^a^
T_2_	274.2^ab^	683.9^b^	1062^a^	2021^b^	330.3^ab^	991^b^	2106	3427	1.205^b^	1.465^b^	1.988^b^	1.702^b^
T_3_	251.2^b^	681.3^b^	1036^b^	1968^c^	310.9^b^	1006^ab^	2092	3408	1.220^c^	1.478^b^	2.018^c^	1.724^c^
SEM	5.30	10.37	4.72	16.56	5.42	11.66	18.66	17.00	0.004	0.005	0.006	0.006
P	0.002	<0.001	0.002	<0.001	0.029	0.016	0.683	0.308	<0.001	<0.001	<0.001	<0.001
Linear P	0.001	<0.001	0.001	<0.001	0.009	0.021	0.566	0.139	<0.001	<0.001	<0.001	<0.001

T_1_: Control; T_2_: Moderate SLAA deficit diet; T_3_: High SLAA deficit diet,

a-c with in a column means bearing different superscripts significantly (p<0.05) differ, SLAA=Second line limiting amino acids, FCR=Feed conversion ratio, FI=Feed intake, BWG=Body weight gain, SEM=Standard error of the mean, SLAA=Second line limiting amino acids

**Figure-1:**
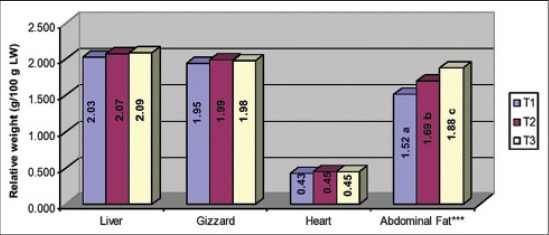
Effect of second line limiting amino acid deficit diets on relative weights of liver, gizzard, heart and abdominal fat of broilers, T_1_- Control; T_2_- Moderate SLAA deficit diet; T_3_- High SLAA deficit diet; *Methionine + Cysteine, ^a-c^Within a column means bearing different superscripts significantly (p<0.05) differ

The fresh carcass weight (Table-5) was significantly (p<0.05) inferior to T_1_ in both treatments with SLAA deficiency. The weight of the breast, thigh, drumstick and wing were significantly (p<0.05) different in T_3_ *vis-a-vis* T_1_ and the corresponding weights in T_2_ were non-significant from both T_1_ and T_3_. The carcass yield, breast yield and thigh yield as percent of live weight were significantly (p<0.05) decreased due to SLAA deficiency and in contrary the drumstick and wing yield remained non-significant. The impact of SLAA deficiency was not evident on the relative weights of internal organs *viz*., liver, gizzard and heart (Figure-1), though the relative weight of abdominal fat was significantly (p<0.05) elevated with SLAA deficiency.

**Table-5:** Effect of second line limiting amino acid deficit diets on carcass characteristics of broilers at the end of 42 days.

Treatment	Carcass cuts (weight in g)					Carcass yield (percent of live weight)				
	
	Fresh Carcass	Breast Weight	Thigh Weight	Drumstick	Wing	Fresh Carcass	Breast Yield	Thigh Yield	Drumstick Yield	Wing Yield
T1	1593^a^	482.0^a^	293.9^a^	222.0^a^	180.5^a^	74.66^a^	22.58^a^	13.76^a^	10.39	8.45
T2	1479^b^	445.3^ab^	265.9^ab^	204.8^ab^	165.7^ab^	73.10^b^	22.00^ab^	13.13^ab^	10.11	8.18
T3	1443^b^	424.6^b^	247.8^b^	192.6^b^	154.0^b^	72.31^b^	21.26^b^	12.40^b^	9.63	7.70
SEM	18.58	7.32	5.40	4.43	4.10	0.239	0.194	0.172	0.153	0.152
P	0.003	0.006	0.002	0.030	0.037	<0.001	0.023	0.005	0.143	0.150
Linear P	0.001	0.001	0.001	0.009	0.011	<0.001	0.007	0.001	0.055	0.059

T_1_: Control; T_2_: Moderate SLAA deficit diet; T_3_: High SLAA deficit diet,

a-c Within a column means bearing different superscripts significantly (p<0.05) differ, SEM=Standard error mean, SLAA=Second line limiting amino acids

The impact of SLAA deficiency on the intake of ME, lysine, sulfur AA (methionine + cystine) and threonine (Table-6) per bird was not evident, while the corresponding measure for CP and SLAA was decreased in a linear (p<0.001) fashion as the level of SLAA deficiency magnified. The absolute intake of valine, isoleucine and tryptophan was significantly (p<0.05) decreased in T_2_ and T_3_ in comparison to T_1_ and further, difference between both T_2_ and T_3_ was quite significantly (p<0.05) noticeable. The decreasing trend in the intake of SLAA was significantly (p<0.001) linear to the extent of the insufficiency. When the intake of these nutrients were expressed to unit weight gain, it was interesting to note that, the ME intake increased linearly to the enormity of SLAA insufficiency. On the other hand, the CP intake per Kg of BWG was incoherent with SLAA deficiency. The intake of lysine, methionine + cystine and threonine per unit of BWG was found to be significantly (p<0.003) amplified to the magnitude of SLAA deficiency. Intake of lysine, methionine + cysteine and threonine per kg BWG in T_3_ was significantly (p<0.05) elevated compared to T_1_, although the difference was non-significant from T_2_. The valine (g/kg BWG) intake in T_2_ was non-significant with control whereas in T_3_ it was significantly lower than T_1_. Considering intake of valine and isoleucine, it was interesting to note that, intake per kg BWG was statistically non-significant in T_2_ *vis-a-vis* T_1_ and the difference was significant only in T_3_ with high degree of SLAA deficiency. The trend of decreasing SLAA intake to the magnitude of SLAA deficiency was highly significantly (p<0.001) linear.

**Table-6:** Impact of second line limiting amino acid deficiency on cumulative absolute and relative intake of ME, CP and Amino Acids.

	ME (M cal)	CP (g)	Lysine (g)	M+C* (g)	Threonine (g)	Valine (g)	Isoleucine (g)	Tryptophan (g)
Intake per bird								
T_1_	10.95	740^a^	36.32	27.68	23.82	29.42^a^	26.25^a^	6.79^a^
T_2_	10.80	713^b^	35.74	27.24	23.47	27.55^b^	24.25^b^	6.43^b^
T_3_	10.74	672^c^	35.53	27.08	23.33	24.82^c^	21.92^c^	5.58^c^
SEM	0.054	7.447	0.175	0.134	0.114	0.476	0.439	0.127
P	0.349	<0.001	0.203	0.219	0.237	<0.001	<0.001	<0.001
Linear P	0.162	<0.001	0.086	0.094	0.102	<0.001	<0.001	<0.001
Intake per kg live weight gain								
T_1_	5.15^a^	348	17.08^a^	13.01^a^	11.20^a^	13.83^a^	12.34^a^	3.19^a^
T_2_	5.35^ab^	353	17.69^ab^	13.48^ab^	11.61^ab^	13.64^a^	12.00^a^	3.18^a^
T_3_	5.46^b^	341	18.06^b^	13.77^b^	11.86^b^	12.61^b^	11.14^b^	2.83^b^
SEM	0.04	2.24	0.14	0.11	0.090	0.157	0.140	0.044
P	0.009	0.103	0.008	0.008	0.007	<0.001	<0.001	<0.001
Linear P	0.003	0.233	0.002	0.003	0.002	<0.001	<0.001	<0.001

T_1_ Control; T_2_ Moderate SLAA deficit diet; T_3_: High SLAA deficit diet;

* Methionine+Cysteine,

a-c Within a column means bearing different superscripts significantly (p<0.05), differ, SLAA=Second line limiting amino acids

## Discussion

The BWG of birds was significantly reduced by SLAA deficiency at all stages of life, which is a clear reflection of impact of treatments on the input of critical nutrients channeled through feed intake and it was found that the bids eat to satisfy the need for a limiting nutrient, and in this attempt the birds might have failed as a result of bulkiness of the feed or the inability of birds to lose sufficient heat to remain in thermal balance, which follows the principles of the Theory of food intake and growth proposed by Emmans [14,15]. The deficiency of SLAA resulted in significantly lower absolute intake of valine and isoleucine (in T_2_) and additionally tryptophan (in T_3_; Table-6) which perhaps impeded protein accretion and hence the growth performance of broilers, since AA have been revealed to influence the myogenic gene expression in broilers [16]. The growth retardation as a consequence to SLAA deficiency is well noticed in previous studies with deficiencies of valine alone in young broilers [17,18], valine and isoleucine [1,19], isoleucine, tryptophan and arginine [20], isoleucine alone [21,22] and arginine, valine, isoleucine and tryptophan [23]. With marginal SLAA deficiency in the finisher phase, birds could compensate to the SLAA intake at this time period (data not shown) and as a consequence, birds could grow on par with control which is suggestive of the compensatory growth during this period due to moderate but not for high SLAA deficiency. The feed intake which was reduced due to SLAA deficiency in the initial pre-starter and starter phases was found to be compensated in the finisher phase suggestive of the adoptive nature of birds to consume more feed owing to relatively well developed intestinal segments at this stage. The non-alteration of cumulative feed intake is largely due to an elevated fatness indicated in SLAA deficit treatments during finisher phase, for obvious reasons discussed above. The reduction of feed intake consequent to the high SLAA deficiency is supported by the studies of [17] with valine, [24] with isoleucine, glycine, glutamic acid and that of [22] with isoleucine deficiencies. In addition, non-alteration of feed intake due to marginal deficiency of valine was reported in finisher broilers [25] is indicative of the adoptive behavior of birds to marginal deficiency of SLAA. The poor FCR of magnitude 0.039 (T_2_) and 0.061 (T_3_) units observed in this study is supportive to the earlier findings with valine (17; 25), with isoleucine, glycine and glutamic acid [24], with isoleucine, tryptophan and arginine [20] and with valine and isoleucine [26,27] deficiencies.

The linear decrease in fresh carcass weight to the magnitude of SLAA deficiency perhaps reflects the response observed for BWG for the reasons discussed previously. The reduction in absolute weight of breast, thigh, drumstick and wing was evident only due to high SLAA deficiency, which is obvious since the requirement of valine is less (76-77% of lysine) for optimum yield of these parameters as compared to the requirement for optimum growth (78% of lysine) as reported by [28]. The carcass, breast and thigh yields were decreased due to high SLAA deficiency and in contrary the drumstick and wing yield remained non-significant which sounds due to relatively less requirement of valine (72-74% of lysine; 28) and probably isoleucine and tryptophan as compared to the requirement for optimum growth. The deficiencies of isoleucine, glycine and glutamic acid [24] and of isoleucine, tryptophan and arginine [20] revealed to significantly alter carcass parameters. In one study [19] the breast meat yield was shown to be more responsive to isoleucine than to valine, which substantiates lesser valine need for breast meat yield. The relative weight of abdominal fat was increased linearly to SLAA deficiency which can be related to the ME intake per kg BWG, which also increased linearly as the SLAA deficiency increased resulting in storage of excess energy in the form of abdominal fat.

The comprehensive intake of ME, lysine, methionine + cysteine and threonine which was similar across the treatments is suggestive of the theory that birds eat to meet the nutrient demands, especially energy because the proportion of lysine, methionine + cysteine and threonine to ME was constant in all the treatments. On the other hand, intake of CP and SLAA were significantly reduced linearly to SLAA deficiency which is obvious as CP and SLAA were reduced in both the treatments. According to the theory of food intake and growth proposed by Emmans [14,15], birds attempt to grow at their genetic potential, which would mean that they attempt to eat sufficient quantity of given feed (hence nutrient) required to grow at that rate. It is clear that, FI or nutrient intake is a function of BWG and hence the individual nutrient intakes were transformed per kg BWG. It was found that ME, lysine, methionine + cysteine and threonine intakes were increased linearly to the magnitude of SLAA deficiency. As the protein or AA content of a diet was reduced, pigs [29-31] and broilers [32,33] found to increase FI to meet their requirement for specific nutrient for potential growth, and the extent to which the nutrient deficiency can be compensated depends on the amount of heat that animal can lose to the environment. This perhaps pertinent to countries like in India where ambient temperature is anticipated to be more than optimal thermo neutral temperature. With this in view, it can be regarded that, excess intake of ME has refrained birds from consuming more feed and hence birds could not eat enough to compensate for the SLAA deficiency particularly in high SLAA deficit diet resulting significant growth retardation.

The RSM although was included 6-10% of ration in previous studies [2-4], yet in all these studies, the CP was made homogenous by adjusting the soybean meal content of the diets. Moreover, in these studies [34,35], for instance at pre-starter phase the total L-lysine level of the ration was maintained at 1.17-1.20% and the threonine (>0.85%) and valine (>1.0%) levels remained excess of requirement for the given lysine levels, thus question of deficiency of third and subsequent limiting AA does not arise, which explains the reason for favorable results with inclusion of 6-10% RSM in previous studies by balancing only for lysine and methionine. In the present study, the lysine level was maintained at 1.33% as recommended for present Cobb broilers, which subsequently increased the requirement of fourth and subsequent limiting AA resulting in a marginal deficiency in RSM based diets and high deficiency in DORB based diets. However, in previous studies, the overall FCR ranged from 1.94 to 2.13 [3] and 2.03-2.30 [4], which was obvious due to low AA density of diets and was incomparable to FCR of the present study as well as FCR of present commercial broiler industry.

The significant depression of bird performance observed in present study with DORB inclusion was in agreement with the previous findings [7,8]. Since, the DORB inclusion is known to reduce the essential AA content of the diet, [34] tried using limiting AA balancing for utilization of used rice bran (with oil) up to 20% of ration. However, the soybean meal content was increased to balance the limiting AA and hence, the CP was increased by 2.00% point in diet balanced for all limiting AA compared to control. In this context, the poor performance observed under 6% RSM and DORB included diets can be clearly attributed to the deficiency of SLAA.

## Conclusion

The study concluded that deficiency of SLAA on RSM and DORB based diets would severely impact the bird performance, carcass parameters and abdominal fat percent depending on the extent of SLAA deficiency. Further studies needed with supplementation of L-valine, L-isoleucine and L-tryptophan to reduce protein in broiler diets by incorporation of RSM and DORB.

## Authors’ Contributions

CBK conceptualized the idea, carried out the experiment, analyzed the data and drafted first manuscript. RGG, KCS, TMP, S and BNS designed and guided during the experiment, helped in analysis of data, given critical inputs and revised the manuscript. GAM helped in carrying out experiment, involved in data collection, laboratory analysis. All authors read and approved the final version of the manuscript.
